# KLK4T2 Is a Hormonally Regulated Transcript from the *KLK4* Locus

**DOI:** 10.3390/ijms222313023

**Published:** 2021-12-01

**Authors:** Åke Lundwall, Erik Bovinder Ylitalo, Pernilla Wikström, Maria Brattsand

**Affiliations:** 1 Translational Cancer Research, Department of Laboratory Medicine, Lund University, SE-223 81 Lund, Sweden; ake.lundwall@med.lu.se; 2 Translational Research Center, Department of Medical Biosciences, Pathology, Umeå University, SE-901 85 Umeå, Sweden; erik.bovinder@umu.se (E.B.Y.); pernilla.wikstrom@umu.se (P.W.)

**Keywords:** kallikrein-related peptidases, KLK4T2, KLK4, KLKP1, prostate cancer, bone metastasis, splice variant, transcripts

## Abstract

The human kallikrein-related peptidase 4 (KLK4) and the transcribed pseudogene KLKP1 are reported to be highly expressed in the prostate. When trying to clone transcripts of KLKP1, we partly failed. Instead, we identified an androgen-regulated transcript, KLK4T2, which appeared to be a splice variant of KLK4 that also contained exons of KLKP1. Expression analysis of KLK4, KLK4T2, and KLKP1 transcripts in prostate cancer cell lines showed high levels of KLKP1 transcripts in the nucleus and in unfractionated cell extract, whereas it was almost completely absent in the cytoplasmatic fraction. This was in contrast to KLK4 and KLK4T2, which displayed high to moderate levels in the cytoplasm. In patient cohorts we found significantly higher expression of both KLK4T2 and KLK4 in benign prostatic hyperplasia compared to both primary prostate cancer and bone metastasis. Analysis of tissue panels demonstrated the highest expression of KLK4T2 in the prostate, but in contrast to the classical KLK4, relatively high levels were also found in placenta. So far, the function of KLK4T2 is still to be explored, but the structure of the translation product indicated that it generates a 17.4 kDa intracellular protein with possible regulatory function.

## 1. Introduction

Prostate cancer (PC) is a major global medical problem, with an annual incidence of close to 1.5 million cases in 2020 (Global Health Observatory) and in Sweden it is the most frequently diagnosed tumor disease among men with about 11,000 new cases per year (Swedish Cancer Registry). PC is a multifaceted disease, and it is not possible today to predict whether a tumor will develop into an aggressive form or not. The mechanisms behind PC metastasis and castration-resistant tumor growth are largely unknown and identification of molecular pathways involved has the potential to improve both PC prognosis and treatment.

The kallikrein-related peptidases, KLKs, are a family of serine proteases, which in humans are encoded by 15 genes situated at a locus on the long arm of chromosome 19 [1,2,3]. Aberrant expression of KLKs has been implicated to be involved in different stages of cancer development. Cleaving molecules and remodeling the extracellular matrix, activation of prohormones, and/or signaling molecules, leads to increased cell proliferation and angiogenesis, which contributes to tumor growth, invasion, and metastasis formation [4,5,6,7,8]. Several studies have also been undertaken to analyze their potential as biomarkers of various cancers, as reviewed in [9,10,11].

We have previously cloned the transcript of KLK3, which perhaps is the most well-known protein in the gene family [12]. It is also known as prostate-specific antigen (PSA) and is currently widely used as a marker for PC detection and disease development. KLK2, which is located next to and is closely related to KLK3, has also been shown to be an excellent PC marker [13]. There are also reports suggesting a role for both KLK2 and KLK3 in the etiology of prostate cancer [14,15]. Another neighbor of KLK2 is KLK4, which was first cloned as enamel matrix serine proteinase 1 (EMSP1) and shown to be important for tooth enamel formation [16]. In parallel, the transcript was cloned and analyzed by other groups under the name prostase, KLK-Like 1, PRSS17, androgen-related message 1 (ARM1), and KLK4 [17,18,19,20]. Study of the gene showed that it was organized with six exons, the first of which containing 5′ nontranslated nucleotides only [19]. There was also a study demonstrating that KLK4 differed from other KLKs, by primarily being synthesized without signal peptide and thus becoming an intracellular protein in the prostate [21].

Between KLK2 and KLK4 on human chromosome 19, there is a gene with the HGNC-approved designation KLKP1. Originally it was identified as a transcribed pseudogene containing a nucleotide sequence homologous with the second translated exon of KLK1 [22]. It has since then been described as a gene generating the noncoding transcript KLK31P, but also of coding transcripts yielding the product KRIP1 [23,24]. These studies also showed that the gene is downregulated during prostate cancer progression.

For studies on the function of and its potential role in PC development, we set out to clone transcripts of KLKP1. During these efforts, we decided to run rapid amplification of cDNA ends (RACE) in order to better understand the transcripts that were predominating in RNA isolated from benign prostate hyperplasia (BPH) tissue and LNCaP cells. To our surprise, the answer was none of the previously described transcripts. Instead we identified a single product, which appeared to be a splice variant of KLK4.

## 2. Results

### 2.1. Identification of a Transcript from the KLKP1 Locus as a Splice Variant of KLK4

Initially, we intended to clone the KLK31P transcripts (GenBank accession number DQ211698 and AY533562) and the transcripts encoding KRIP1 (GenBank accession number DQ086829), using RNA isolated from BPH tissue and LNCaP cells as template for RT-PCR. Despite repeated efforts, we failed to generate the KLK31P transcripts. We were more successful with the KRIP1 transcript, but in addition to the expected transcript, the RT-PCR also generated a smaller product in which nucleotides 358–787 of DQ086829 were missing. As there were GT and CAG sequences at the ends of the missing piece, it most likely was removed by a splicing event, which also, almost completely, removed the nucleotides coding for KRIP1.

**Figure 1:**
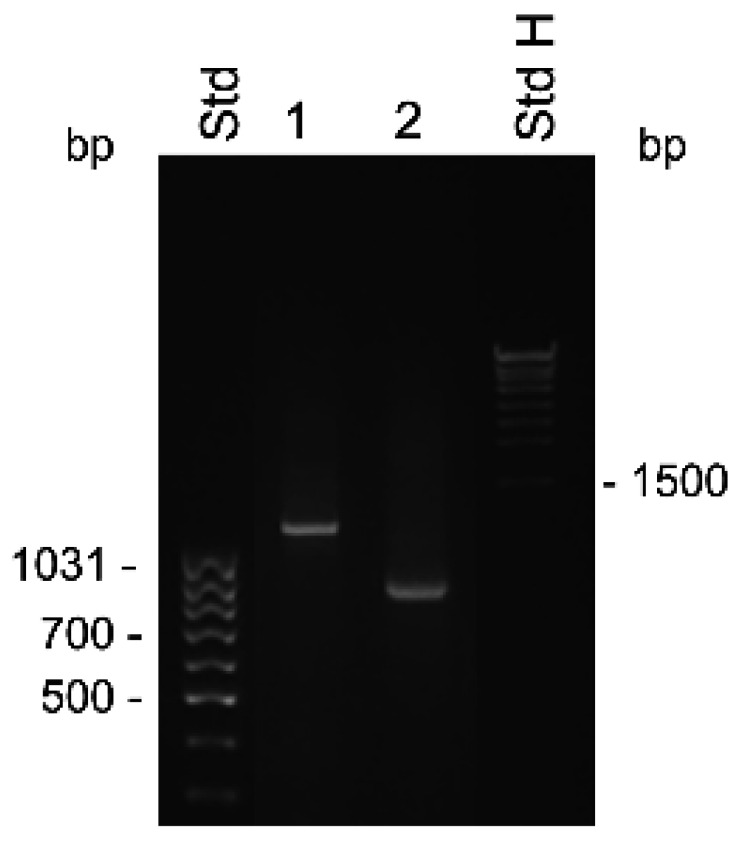
Detection of KLK4T2 by RACE. RACE products generated from 2 ng unfractionated LNCaP cell RNA were analyzed by electrophoresis on 1% agarose gel. Lane 1 shows 5′ RACE with the specific primer KLKP9R and lane 2 shows 3′ RACE with the specific primer KLKP8F. For information about the primer sequences, see Table 1. MassRuler Low Range (Std L) and High Range (Std H) were used as molecular size markers. Relevant sizes in bp are indicated in the figure.

The difficulty in obtaining the KLK31P transcripts in combination with our discovery of a new transcript from the KLKP1, prompted us to reinvestigate the products of the gene by RACE analysis. We used the primer KLKP9R, originally synthesized for cloning of KLK31P and KRIP1, in 5′ RACE experiments (Table 1). To rule out heterogeneity in the 3′ end, we also made 3′ RACE with an oligonucleotide, priming to a common sequence of KLK31P and KRIP1, which is also homologous with exon 2 of KLK1. The results of the RACE experiments are shown in Figure 1. The 3′ RACE yielded a single 0.8 kb product, with a nucleotide sequence identical to previously reported transcripts, and ending in a poly(A) tail showing it was derived from an mRNA. The 5′ RACE yielded a single product of 1.2 kb, suggesting a single predominating product from the gene. The nucleotide sequence of the 3′ part of the transcript was identical to the one we generated by 3′ RACE. Upstream of the sequence homologous with the second exon of KLK1, we expected to find the sequence present in both KLK31P and KRIP1. To our surprise, this was not the case. Instead there was a sequence that we could identify as coming from exon 4 and 3 of KLK4 and with a 5′ terminus that was very similar to the one reported for the transcript of the intracellular form of KLK4 [21]. Thus, the major product of the KLKP1 locus seemed to be a splice variant of KLK4. We named this transcript KLK4T2.

**Figure 2:**
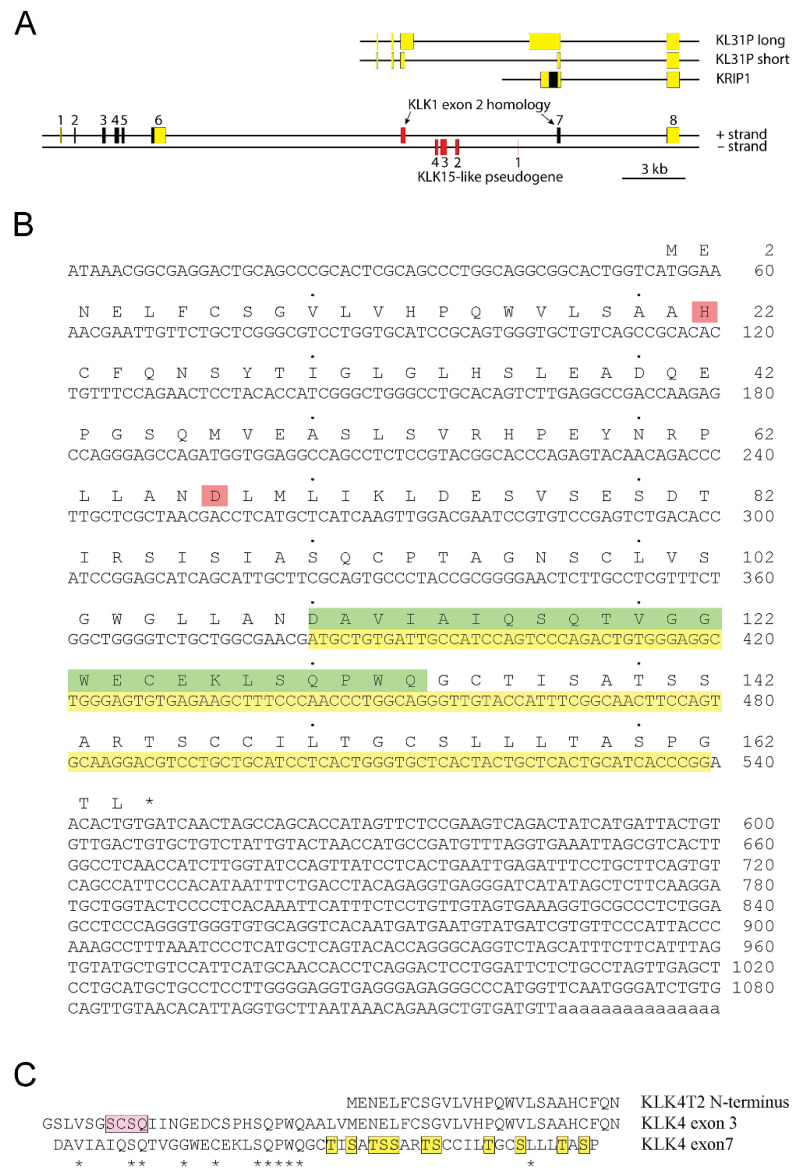
The extended KLK4 locus. (**A**) Schematic drawing of the KLK4 gene with location of previously reported KLKP1 genes illustrated above. Exons are indicated by boxes with translated sequences shown in black and nontranslated sequences in yellow. Exons of pseudogenes are colored in red. The new KLK4T2 transcript is formed by exon 4, 7, 8, and part of exon 3. (**B**) The cDNA sequence of KLK4T2 is shown with translation in one letter code written above. Stop codon is marked with an asterisk. The His and Asp part of the catalytic triad in KLK4 are marked in pink. Nucleotide sequence homologous to KLK1 exon 2 is highlighted in yellow, and the corresponding amino acid sequence until the frame shift, in green. (**C**) Conservation of amino acid residues encoded by exons 3 and 7 in KLK4. Amino acid alignments showing conserved residues encoded by KLK4 exon 3 and 7 indicated by stars. The pro-piece of proKLK4 is highlighted in pink and the Ser and Thr residues in the C-terminal part of the KLK4T2 product are highlighted in yellow. The N-terminal part of the KLK4T2 product is shown above the aligned sequences for comparison.

Our result also suggested that KLK4 was a fairly large gene, consisting of at least 8 exons (Figure 2A). The structure of KLK4T2 cDNA with its translation is shown in Figure 2B. The transcript did not encode a signal peptide and therefore, the product presumably was a small intracellular protein with a predicted molecular mass of 17.4 kDa. The His and Asp of the catalytic triad were preserved in the KLK4T2 product, which most likely was lacking proteolytic activity, as the catalytic Ser, located in exon 5 of the gene encoding the KLK4 protease, was missing.

An interesting observation was made when aligning the primary structures encoded by exon 3 and exon 7 of the extended KLK4. There were conserved residues that reached approximately halfway into the exons, as seen in Figure 2C. This region of the KLK4 protease holds the residues of the pro-piece and the amino terminus of the activated protein. The reminder of exon 7 encoded a region rich in Ser and Thr, whereas in exon 3 it was identical with the amino terminus of the KLK4T2 product.

### 2.2. Analysis of Transcripts from the KLK4/KLKP1 Locus by RACE

**Figure 3:**
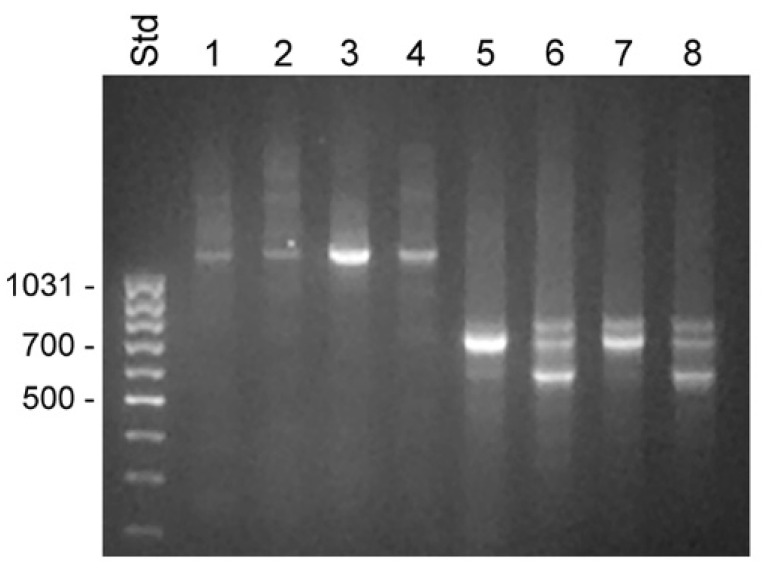
Transcripts detected by 5′ RACE in unfractionated BPH and compartments of LNCaP cells. RACE products were analyzed by electrophoresis in 1% agarose. Lanes 1–4 show 5′ RACE experiments using the KLK4T2-specific primer KLKP9R and lanes 5–8 using the primer KLK4r18, specific for classical and truncated KLK4. Products generated with unfractionated RNA from BPH are shown in lanes 1 and 5 and from LNCaP in lanes 2 and 6. Products generated with RNA from the cytosol of LNCaP are shown in lanes 3 and 7, and from the nucleus in lanes 4 and 8. MassRuler Low Range (Std) was used as size marker and relevant sizes in bp are written to the left.

The 5′ RACE experiments were run on samples made with total RNA from BPH tissue and LNCaP cells and fractionated RNA isolated from the cytosol and nucleus of LNCaP cells. Priming with oligonucleotide KLKP9R, which hybridized to the 3′ end of KLK4T2 and also of KLK31P and KRIP1, yielded a single predominating product of 1.2 kb in all samples (Figure 3). Sequencing of the product showed that it was identical to KLK4T2.

The primer KLK4r18, which hybridized to a sequence immediately upstream to the stop codon in KLK4, resulted in a more complex pattern, with a 0.7 kb product that was present in all samples (Figure 3). In the BPH sample, it appeared as a single predominating product, but it was also the major product in the sample from LNCaP cytosol. DNA sequencing identified it as a KLK4 transcript, identical to that described by [21], with a 5′ end very similar to that of KLK4T2, but around 30 bp shorter. The dominating component in LNCaP samples made from nucleus or total RNA was a 0.6 kb product. The sequencing of the cDNA showed that it had the expected 3′ end in exon 6, but then the sequence continued into intron 5, where it after 319 bp ended in a polyA tract. A third component was also evident in all LNCaP samples. The cDNA sequence of this 0.8 kb product showed it to be identical to the 0.7 kb component, but also carrying the fourth intron of KLK4, which had not been spliced out. Both the 0.6 and 0.8 kb products could indicate that the splicing machinery in the LNCaP cells is less efficient.

**Figure 4:**
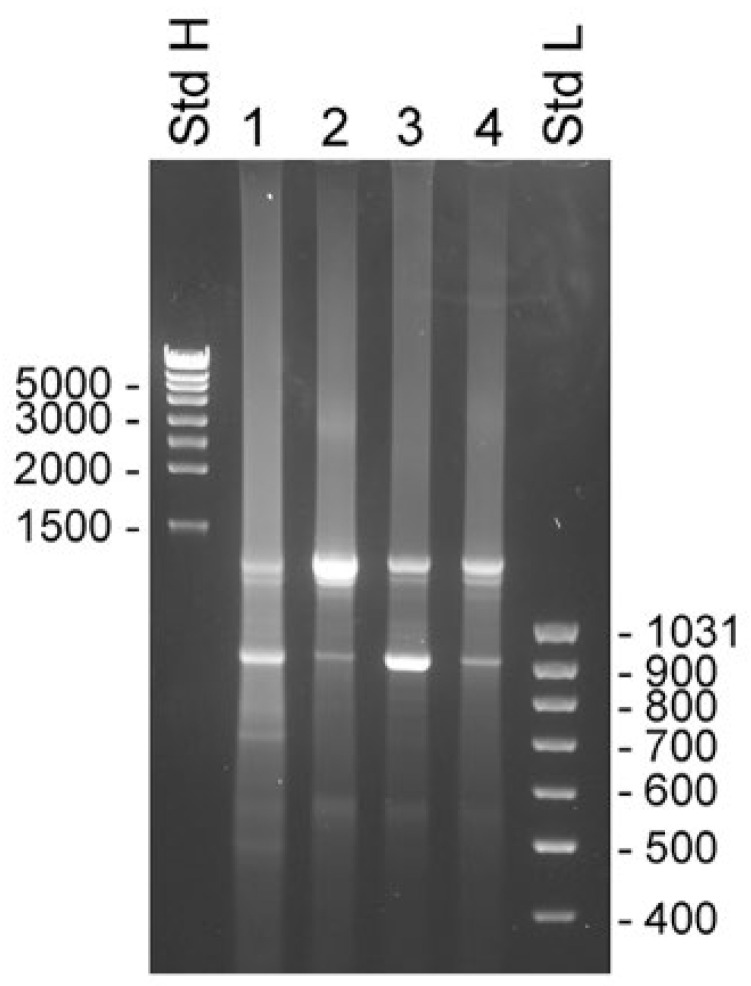
Transcripts detected by 3′ RACE in unfractionated BPH and compartments of LNCaP cells. RACE products were analyzed by electrophoresis in 1% agarose gels. Experiments were done with the primer KLK4Ne3f, which is specific for KLK4, exon 4, which will generate product from both classical and truncated KLK4, and KLK4T2. The 3′ RACE-ready cDNA was synthesized with unfractionated RNA from human BPH tissue (lane 1) and LNCaP cells (lane 2), and RNA from LNCaP cytosol (lane 3) and nucleus (lane 4). MassRuler high (Std H) and MassRuler low (Std L) were used as size markers. Relevant sizes in bp are indicated in the figure.

The 3′ RACE experiments were made with samples taken from the same sources as used for the 5′ RACE experiments. The primers used were KLK4Ne2f and KLK4Ne3f, which hybridized with sequences in KLK4 exon 3 and 4, respectively. Both primers gave very similar results, with the exception that priming in exon 3 yielded slightly larger products that reflected the 262 bp difference in location of priming sites (Figure S1). There were two major components present in all samples. The estimated molecular sizes of products generated by KLK4Ne3f were 1.3 and 0.9 kb (Figure 4). The cDNA sequence showed that the smaller product emanated from KLK4T2, which was predominant in samples made with total RNA from BPH and LNCaP cytosol. The larger product was predominant in samples made with total RNA and the nucleus fraction of LNCaP cells. The sequence of the product generated from total RNA of LNCaP yielded a sequence extending from exon 4 to exon 5 and then continued into intron 5, where it ended in a poly(A) sequence located 935 bp into the intron. Sequencing of the upper band from BPH prostate and cytosol of LNCaP yielded unreadable sequences, presumably due to heterogeneity of the 3′ RACE products.

**Figure 5:**
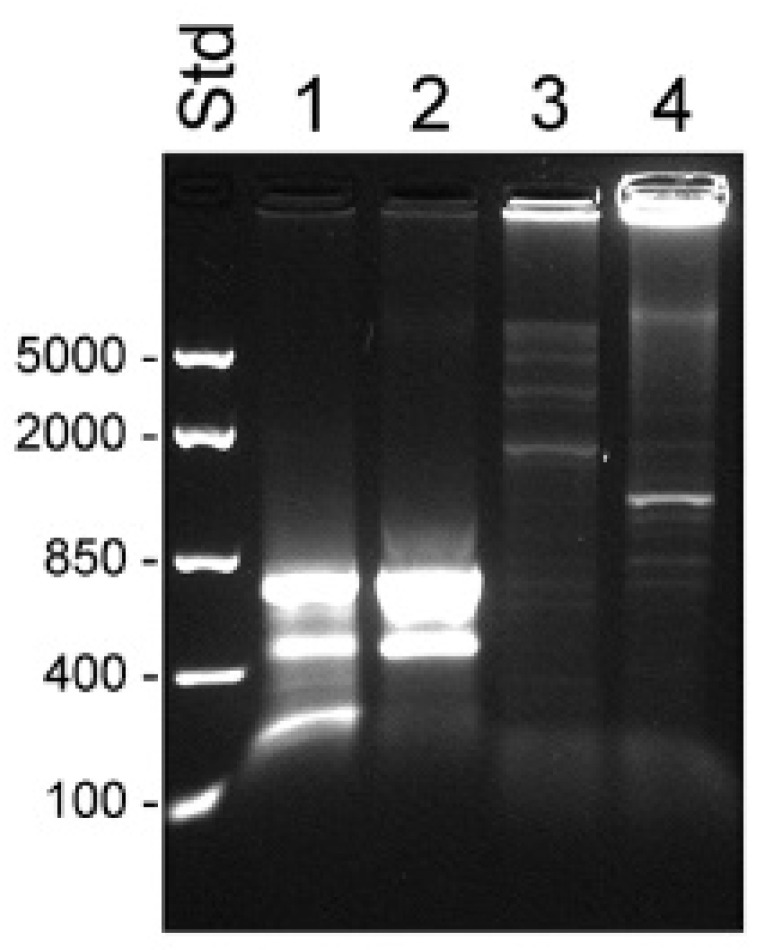
Transcripts detected by 5′ RACE PCR in RNA from prostate cancer bone metastasis. RACE products were separated on a 1.2% agarose gel. The 5′ RACE cDNA was synthesized using pooled total RNA prepared from bone metastasis of patients suffering from castration-resistant (lanes 1 and 3, *n* = 5) or hormone-naïve (lanes 2 and 4, *n* = 2) prostate cancer. PCR was run using KLK4-specific oligo KLK4r18 (lanes 1 and 2) or KLK4T2-specific oligo KLKP9R (lanes 3 and 4) in combination with the UPM primer from the RACE kit. RNA was prepared using AllPrep DNA/RNA/Protein kit (Qiagen). FastRuler Middle Range (Std) was used as molecular weight standard, and relevant sizes in bp are indicated in the figure to the left.

The 5′ RACE cDNA was also synthesized from pooled material consisting of total RNA prepared from bone metastasis of patients suffering from castration-resistant prostate cancer (CRPC) or hormone naïve prostate cancer (HNPC) (Figure 5). Using the KLK4-specific primer KLK4r18, three products were formed from the CRPC and two from the HNPC material (lanes 1 and 2). The largest, 0.7 kb product, corresponded well with the sequence published by Korkmaz et al. [21], starting 20–40 bases into exon 3, followed by exons 4–6. Some of the fragments contained an extra 12 bp insertion between exon 3 and 4. The 0.6 kb product seemed to be a splice variant resembling the larger product except it totally lacked exon 5. The third and smallest band from the CRPC turned out to be a mix of primer dimer and nonidentified short sequences. Several diffuse, high molecular weight bands were formed when using the KLK4T2-specific primer KLKP9R in 5′ RACE PCR using CRPC as template, but no clones could be isolated and sequenced from this material (Figure 5, lane 3). When using HNPC as template, a weak 1.2 kb fragment was formed that corresponded to KLK4T2. In the CRPC sample, but especially when using HNPC as template, we also obtained amplification of fragments so large that they could hardly enter the gel (Figure 5, lanes 3 and 4).

### 2.3. Analysis of Transcripts from the KLK4/KLKP1 Locus by RT-qPCR

**Figure 6:**
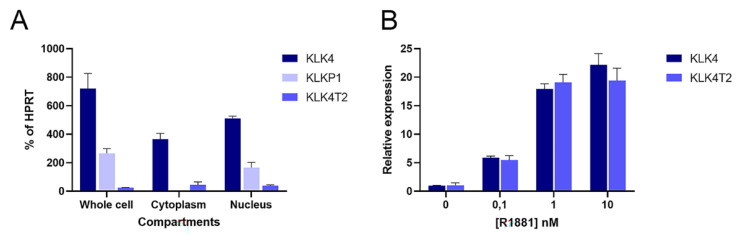
Cellular location and androgen stimulation of transcripts. Transcripts were measured by RT-qPCR in extracts of LNCaP cells. PCR was run using the primer pairs KLK4Ne3f-KLK4Ne4r amplifying KLK4, and K4T2e23f-K4T2e3r specific for KLK4T2. KPT2e1f-K4T2e3r used for KLKP1 amplifies both KRIP1 and KLK31P long transcripts. The results are given as concentration relative to the transcript concentration of the housekeeping gene *HPRT*, which was amplified using HPRTf1-HPRTr2. The error bar represents one standard deviation. (**A**) The relative amounts of KLK4, KLK4T2, and KLKP1 transcripts in total (whole cell compartment) or fractionated RNA (Cytoplasm or Nucleus) are shown. (**B**) To explore the androgen sensitivity, RT-qPCR was run on RNA prepared from LNCaP cells induced with 0.1, 1, and 10 nM R1881, as indicated in the figure. The amount of KLK4 and KLK4T2 transcript are expressed relative to noninduced levels for each species.

Because of the discovery in the RACE experiments, we decided to quantify transcripts from different compartments of the combined KLK4 and KLKP1 loci. We used primers that would specifically recognize KLK4, KLK4T2, and KLKP1 (KRIP1 and the longer form of KLK31P) in RT-qPCR experiments. This showed that the transcripts encoding KLK4 and KLK4T2 were found in the cytosol fraction (Figure 6A). In contrast, the KLKP1 transcripts were enriched in the nuclear fraction, while it was more or less eliminated in the cytoplasmatic fraction.

Hormone effect on the KLK4T2 expression was studied in androgen-stimulated LNCaP cells. The androgen stimulation resulted in an increase of KLK4T2 transcripts, which was of a similar magnitude as the hormone-induced increase of KLK4 (Figure 6B).

### 2.4. Expression Pattern of KLK4-Related Transcripts in the Prostate and during Prostate Cancer Disease Progression

**Figure 7:**
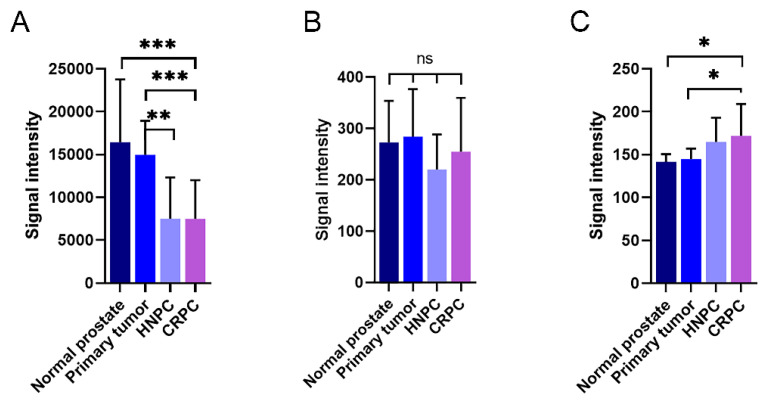
Expression of KLK4-related transcripts in patient materials measured using an Illumina whole genome expression array. The amount of KLK4-related transcripts designated as KLK4 (**A**) and KLKP1 (**B**) were analysed in normal prostate, primary prostate cancer tumours, and bone metastasis from patients suffering from hormone-naïve (HNPC) or castration-resistant prostate cancer (CRPC), respectively. The corresponding results for the proliferation marker Ki67 are shown in (**C**). The error bars represent one standard deviation. *** *p* < 0.001, ** *p* < 0.01, * *p* < 0005, ns = not significant.

In Illumina expression array analysis, two different probes were hybridizing with KLK4-related transcripts, denoted KLK4 and KLKP1. As can be seen in Figure 7A, the expression of KLK4 was very high in both normal and primary prostate cancer tissue. When the disease progressed, the transcript levels of KLK4 were significantly reduced, as can be seen from the levels in both hormone-naïve and castration-resistant bone metastases. This was in contrast to the findings for KLKP1, where no significant differences could be observed between the different disease stages (Figure 7B). Contrary to KLK4, the Ki67 expression, representing tumor cell proliferation rate, was as expected, positively correlated to the disease stage (Figure 7C).

**Figure 8:**
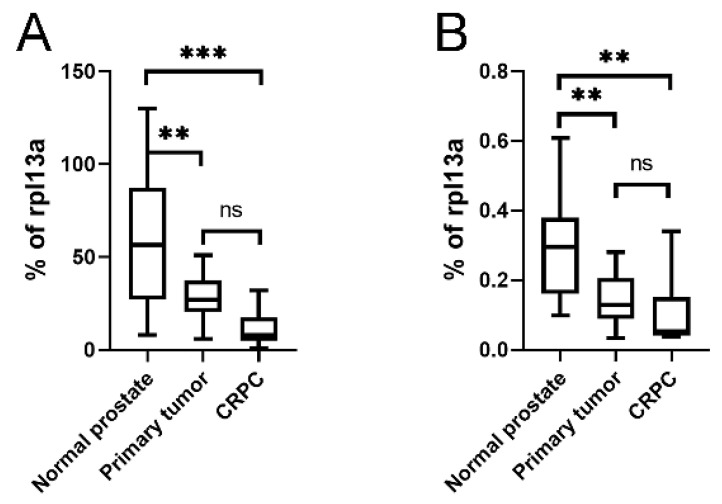
Expression of KLK4 and KLK4T2 transcript in patient material. Transcript levels were measured by RT-qPCR on unfractionated RNA prepared from normal human prostate tissue (*n* = 10), primary PC tumor (*n* = 13) or bone metastasis from CRPC patients (*n* = 9). In the qPCR, the primer pairs KLK4Ne3f-KLK4Ne4r amplifying KLK4 (**A**) and K4T2e23f-K4T2e3r specific for KLK4T2 (**B**) were used. The results are given as concentration relative to the transcript concentration of the housekeeping gene rpl13a, which was amplified using primer pairs rpl13a-f–rpl13a-r. Samples were run as triplicates, and data are plotted as boxplots with the median value as a line across the box and the first quartile (Q1) value at the bottom and the third (Q3) at the top. The whiskers show the highest and lowest values in each group. *** *p* < 0.001, ** *p* < 0.01, ns = not significant.

As the KLKP1 probe used in the Illumina array showed 100% sequence identity with both KLK4T2 as well as several other published KLKP1 variant sequences, we went on and ran RT-qPCR on patient samples using primer pairs specific for KLK4 and KLK4T2. In Figure 8, the expression pattern in normal prostate tissue (*n* = 10), primary tumor tissue (*n* = 13), and bone metastasis from CRPC (*n* = 9) are displayed. As can be seen, the expression of KLK4 was significantly decreased as the disease progressed, although the most significant difference is seen as the disease progressed into bone metastasis (Figure 8A). The expression of KLK4T2 (Figure 8B) was also decreased in primary tumor and bone metastasis compared to normal tissue.

### 2.5. Tissue Expression

**Figure 9:**
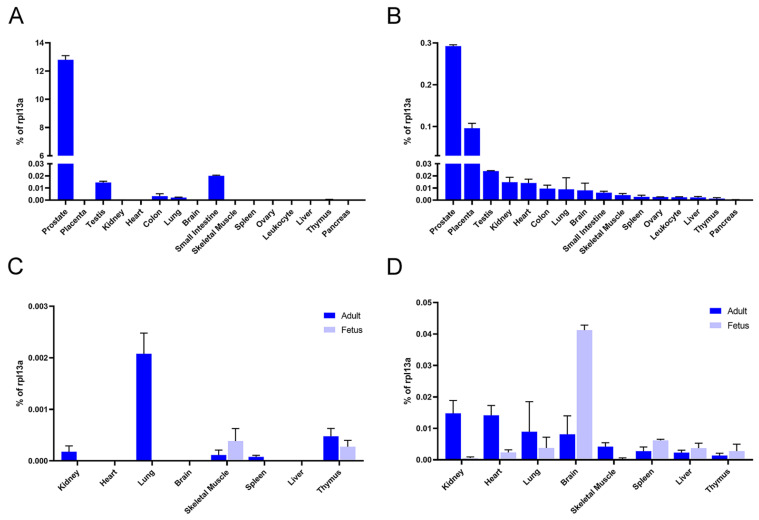
Expression of KLK4 and KLK4T2 in different tissues. qPCR was run on MTC panels using the primer pairs KLK4Ne3f-KLK4Ne4r amplifying KLK4 (**A**,**C**) and K4T2e23f-K4T2e3r specific for KLK4T2 (**B**,**D**). The results are given as concentration relative to the transcript concentration of the housekeeping gene *rpl13a*, which was amplified using rpl13a-f–rpl13a-r. Samples were run as triplicates, and error bars indicate one standard deviation. In (**A**,**B**), expression is shown in human adult tissue, whereas in (**C**,**D**) expression in fetal tissue has been included as indicated in figure legends.

In order to examine the expression profile in tissues of the new transcript, we set out to run qPCR on cDNA from different MTC panels. We used primer pairs specific for KLK4T2 and also included the results from the expression of the classical KLK4 transcript as comparison. As can be seen in Figure 9, the highest expression of KLK4T2 was found in the prostate, although the levels were not as high as those for KLK4 transcript. In contrast to KLK4, however, KLK4T2 seemed to be less restricted in expression and relatively high levels were also found in the placenta, followed by testis, kidney, and heart. KLK4 showed similar expression levels in testis as KLK4T2, but no expression at all could be seen in the placenta, kidney, or heart. The comparison becomes even more interesting when including fetal tissues. As can be seen in Figure 9C,D, the highest expression in fetus was from KLK4T2 in brain tissue, whereas no expression of KLK4 could be detected in either adult or fetal brain. Please note the difference in *y*-axis scales in Figure 9C,D.

## 3. Discussion

In this paper, we describe a transcript, KLK4T2, encompassing nucleotide sequences from both the KLK4 and the KLKP1 loci on human chromosome 19. Translation of the transcript showed that it encoded a small 17.4 kDa protein with a very interesting structure (Figure 2). There is no signal peptide sequence in the N-terminal end, indicating that it encodes an intracellular protein, and in the C-terminal end the sequence is rich in Ser and Thr—residues that may be phosphorylated and thereby hints to that the KLK4T2 product perhaps could be a signaling molecule. Furthermore, the conservation of amino acid residues encoded by the 5′ halves of exon 3 and 7 was intriguing, as it suggests that the exon-7-derived residues may complement the KLK4T2 product with a structure resembling the part of pro-KLK4 encoded by the 5′ end of exon 3 (Figure 2C). Perhaps, would this structure also mimic the 3D structure of pro-KLK4 in this part of the molecule, which holds both the N-terminus of KLK4 and the pro-piece of the precursor? Following four fully conserved amino acid residues approximately halfway into the exon, there was a shift of reading frame in exon 7, leading to a loss of KLK structure for another structure rich in Ser, Thr, and Cys. We would be very surprised if this beautiful structure had developed by mere coincidence.

The nucleotide sequence of KLK4T2 and the two other transcripts described for KLK4 [16,21] strongly indicates that the gene must have at least two transcription initiation sites. Upstream to the translation start site of KLK4 protease, there is an appropriately located TATAA sequence, which is present in the upstream promoter of many genes, to direct the formation of the preinitiation complex and subsequent RNA polymerase II binding and mRNA synthesis [19]. There was no TATA-box immediately upstream to the transcription start site of KLK4T2. Instead there was a region rich in C and G nucleotides, with a frequent occurrence of CpG sites and SP1 recognition sequences. Such structures are commonly associated with promoters of housekeeping genes and other low expressed genes.

During our investigation of KLK4T2, we discovered a paper on chimeric sense-antisense transcripts emanating from the KLK4 gene [25]. In that paper, they describe a transcript that appears to be very similar, if not identical, to KLK4T2. They suggest that the transcript is created by trans-splicing of KLK4 and KLKP1 transcripts, but without any supporting experimental data. In a more recent article by another group, it was described as a transcript generating a KLK4–KLKP1 fusion protein [26]. The amino acid sequence of the fusion protein is identical to that of the KLK4T2 protein product, but the transcript sequence (Genbank MN037411) includes the full sequence of exon 3 and is 32 bp longer than KLK4T2 described in this paper. There was no information in the paper as to how the sequence was obtained.

In the light of our experiences, while trying to generate KLKP1 transcripts and the following RACE experiments resulting in the identification of KLK4T2, we began to suspect that KLKP1 transcripts may not be mature. The RT-qPCR experiments showed that the KLKP1 transcripts were absent in the cytoplasmatic RNA fraction (Figure 6A). This was in stark contrast to the major KLK4 transcript and KLK4T2, which both were present in the cytosol at a high to moderate level. These results indicated that the KLKP1 transcripts very likely are immature products of partly spliced KLK4. However, the KLKP1 transcripts have also been detected by Northern blotting. Both Lu et al. and Kaushal et al. detected hybridizing RNA in the prostate, yielding a strong signal with a molecular size of 1.6 kb [23,24]. They both also detected a weaker hybridizing material in RNA from pancreas with a size of 1.2 or 1.1 kb. Unfortunately, both groups used probes that were overlapping with what we in this article describe as exon 7 of KLK4, which shows 80% sequence similarity with exon 2 of KLK1 and 75% with exon 2 of KLK2 and KLK3. The latter, which codes for PSA, is one of the most abundant transcripts in the prostate, as is also KLK2. The size of the transcript is just below 1.5 kb for both KLK2 and KLK3, but with a poly(A)-tail attached they will probably be around 1.6 kb. Therefore, if the stringency of hybridization and washings are not carefully controlled, it could very well be that it is KLK2 and KLK3 that generate the signal in the prostate, and not KLK31P or KRIP1. Kaushal et al., using a labelled cRNA probe for the experiment, performed a simultaneous hybridization to a dot blot with 15 different KLK cDNA, including those of KLK2 and KLK3, to keep control of cross-hybridization. However, because of differences in melting temperature, i.e., stability of hybrids, the RNA:DNA hybrids could have been washed away under very stringent conditions where RNA:RNA hybrids remain stable. A probe lacking exon 7 could have been used in order to avoid potential cross-hybridization.

In the paper of Chakravarthi et al. [26], they refer to KLK4 as a protein overexpressed in prostate cancer, and KLKP1 as a transcript only expressed in normal prostate, and they claim that their fusion transcript KLK4–KLKP1 would be prostate cancer specific. This is in contrast to the findings we have in this paper, where we show that the expression of both KLK4 and KLK4T2 is reduced in prostate primary tumors and metastasis (Figure 7 and Figure 8). Moreover, we find our KLK4T2 transcript expressed in normal prostate as well as some other tissues, and we could also see expression in some tissues at the fetal stage (Figure 9).

Thus far, we have only been able to detect the protein in recombinant form when expressed in bacteria, not in eukaryotic cells. This could of course be due to the fact that no KLK4T2 protein is produced, but another explanation could be that it is only formed during certain conditions that we have not yet tested. It is well known that many regulatory proteins have a very short half-life, and this in combination with a moderate expression level could result in protein levels that are below the detection limit of our antibodies (data not shown). As mentioned above, the structure contains both several possible phosphorylation sites and a putative cleavage site, which together indicate a protein with high turnover rate.

There are several studies reporting that the expression of KLK4 is higher in prostate tumors compared to normal, nonmalignant prostate tissue [27,28,29,30]. As can be seen in Figure 7 and Figure 8, this is in contrast to our results where the expression of KLK4 is much lower in bone metastasis than in normal prostate tissue or primary tumors. Part of this could be due to that the bone metastasis also contains nonepithelial cell types that have no expression of KLK4 transcript, but it is also well known that the expression of KLK3, for example, decreases as the tumor cells become less differentiated [31], and the expression levels are much lower in PC cell lines than in the patient material (data not shown). In contrast to KLK4 (Figure 7A), no significant reduction can be seen in the KLKP1 signal as the disease progresses (Figure 7B), which indicates differences in the regulation of the expression. As mentioned earlier, the KLKP1 probe is homologous to several alternatively spliced transcripts from the KLK4 gene locus, including KLK4T2, KLK31P long, KLK31P short, and KRIP1. As shown in Figure 8, the levels of KLK4T2 transcripts are reduced as the disease progresses into the metastatic phase. As the Illumina array is run on total RNA prepared from the patient samples and not the cytoplasmic fraction, the contribution from other KLKP1 transcripts is probably the reason for no significant difference seen between normal prostate tissue and more advanced stages of prostate cancer in the Illumina array. When comparing, the expression of KLK4T2 is about 100-fold lower than the extremely high expression of KLK4. Still, it is expressed at 10-fold higher levels than the other KLKP1 variants taken together (data not shown).

KLK4 was originally isolated, characterized, and cloned from porcine transition stage enamel matrix, under the name enamel matrix serine proteinase 1 [16]. The cDNA sequence coded for the precursor of a typical simple serine proteinase, with a signal peptide and a pro-piece. Later, KLK4 transcripts were identified in the human prostate and a new truncated transcript was also described [21,32]. Our results confirm that a truncated form of KLK4 is the predominating molecular species in BPH, PC bone metastasis, and cell lines (LNCaP in this paper; 22Rv1, VCaP, and LNCaP C4-2B data not shown). The product of this transcript is lacking a signal sequence and would therefore most likely be an intracellular protein (Figure 2). Furthermore, the conserved hydrophobic N-terminus, present in all serine proteases, which following activation aids in the formation of a functional catalytic pocket was also missing. It is therefore very unlikely that the truncated KLK4 transcript would generate a functional protease. We have had problems in detecting this truncated protein using both in-house-produced KLK4-specific antibodies and commercially available KLK4 antibodies (data not shown). This is in sharp contrast to the case with KLK3, where high levels of transcript and protein are readily detectable. In the Illumina expression arrays, we found that the transcript levels of KLK4 were at the same high levels as those for KLK3 (data not shown). Considering the discrepancy between the high levels of KLK4 transcripts found and the difficulty in identifying the protein, it could be speculated that the truncated KLK4 expressed in the prostate, instead of being a coding RNA could be a regulatory RNA.

## 4. Materials and Methods

### 4.1. Nomenclature

In this paper, we identified a new splice variant of the KLK4 locus that we denote KLK4T2 (OK423452). KLK4 is mentioned as either classical KLK4, which refers to pre-pro-KLK4 with signal peptide and pro-peptide (NM_004917) [16,20], or truncated KLK4, which is lacking the signal sequence, pro-peptide, and N-terminus of pre-pro-KLK4 [21]. KLK4 without specification refers to both forms of KLK4, with the exclusion of KLK4T2. Numbering of KLK4 exons starts with the 5′ nontranslated first exon identified by Hu et al. [19]. This first exon was not included in Nelson et al. [20], resulting in a discrepancy in numbering of exons.

### 4.2. Cell Culture

LNCaP (ATCC CRL-1740) were maintained in RPMI 1640 + GlutaMAX supplemented with 10% fetal bovine serum (FBS), 100 U penicillin/mL and 100 µg streptomycin/mL, 10 mM HEPES, and 1 mM sodium pyruvate (GIBCO, Thermo Fisher Scientific (Stockholm, Sweden)). Cell cultures were performed at 37 °C in a humidified atmosphere containing 10% CO_2_.

### 4.3. RACE PCR

For amplification of 5′ and 3′ ends of the KLK4 transcripts, the SMARTer 5′/3′ RACE kit (Clontech, TakaraBio (Saint-Germain-en-Laye, France)) was used. Products were analysed by agarose gel electrophoresis, stained with GelRed (SigmaAldrich (Stockholm, Sweden)), and visualized by UV light. Larger quantities of products were obtained by reamplification of specific bands on the agarose gel. For this, approximately 1 µL was recovered directly from the visualized band in the agarose gel and diluted in 15 µL H_2_O, of which 1 µL was used as template in 50 µL PCR reaction using gene-specific primers in combination with the universal primer mix (UPM) supplied with the SMARTer 5′/3′ RACE kit (Clontech, TakaraBio (Saint-Germain-en-Laye, France)). The PCR product was purified using the Qiaquick PCR purification kit (Qiagen (Kista, Sweden)) and the DNA concentration was determined by measurement of the absorbance at 260 nm. The yield of product was usually in the range 1–10 µg. The PCR products were sequenced using the BigDye Terminator v3.1 Cycle sequencing kit and an ABI PRISM 3100 Genetic Analyzer (Applied Biosystems, ThermoFisher Scientific (Stockholm, Sweden)), following the recommendations of the supplier of the kit. DNA sequencing was also made at GATC Biotech, currently Eurofin Genomics (Solna, Sweden). Alternatively, RACE products were isolated from agarose gels by purification of excised bands using the Macherey–Nagel™ NucleoSpin™ Gel and PCR Clean-up kit (Düren, Germany) according to manual. Transcripts were cloned into either the pRACE vector from the SMARTer kit 5′/3′ RACE kit (Clontech, TakaraBio (Saint-Germain-en-Laye, France)) or the pcDNA3.1/V5/His expression vector (Invitrogen, Thermo Fisher Scientific (Stockholm, Sweden) and the inserts were DNA sequenced as described above. Primers used are listed in Table 1.

### 4.4. RNA Preparation, RT-PCR, and cDNA Cloning

Total RNA was prepared using either the AllPrep DNA/RNA/Protein kit (Qiagen (Stockholm, Sweden)) or the Ambion Paris kit (Thermo Fisher Scientific (Stockholm, Sweden)). Cytosolic and nuclear RNA was prepared from patient samples using SurePrep Nuclear or Cytoplasmic RNA Purification Kit (FisherScientific GTF (Gothenburg, Sweden)). The Ambion Paris kit was used for fractionation of RNA from LNCaP cells. cDNA was synthesizes using either Revert Aid or SuperScript VILO (both Thermo Fisher Scientific (Uppsala, Sweden)). DNA was amplified using BioRad iQmix (Solna, Sweden) for quantification or either Advantage 2 (Clontech (TakaraBio (Saint-Germain-en-Laye, France)) ) or Platinum Hot Start PCR mix (Invitrogen, Thermo Fisher Scientific (Stockholm, Sweden)) for visualization and/or cloning. For primer sequences, see Table 1.

### 4.5. Patient Samples and Gene Expression Analysis

Whole genome expression analysis of nonmalignant and malignant areas of prostatectomy samples (*n* = 13) and of hormone-naïve (*n* = 10) and CRPC (*n* = 30) bone metastases samples were previously performed based on Illumina bead arrays, as described in [33]. From these data, relative expression levels of KLK4, KLKP1, and proliferation marker Ki67 were extracted. For a subset of those samples, with RNA extracts available, RT-PCR analysis of KLK4-related transcripts was performed. For expression analysis in different organs, qPCR was run on MTC Panels I and II and fetus from Clontech, TakaraBio (Saint-Germain-en-Laye, France)). See figure captions and Table 1 for information about primer pairs used.

### 4.6. Statistical Analysis

One-way ANOVA was used to show that we had significant differences between our groups. The ANOVA study was followed by Tukey’s HSD Test for multiple comparisons.

### 4.7. Accession Numbers

KLK4T2 received GenBank accession number OK423452.

## 5. Conclusions

We identified an exciting new transcript, KLK4T2, formed by alternative splicing of the KLK4 gene. The transcript is regulated by androgens and primarily high levels in the prostate, but also in the placenta and fetal brain. KLK4T2 is, in contrast to KLKP1 transcripts, expressed in the cytoplasm of the cell, a prerequisite for translation to occur. No signal sequence could be identified in the amino acid sequence, indicating a nuclear location of the protein. Several putative phosphorylation sites and a proposed cleavage site open up for a regulatory protein with fast turnover rate.

## Figures and Tables

**Table 1:** Primers used for PCR, cloning, and DNA sequencing. Bases written in lower case are non-sequence-specific nucleotides included for the cloning procedure.

Primer	Sequence 5′–3′	Purpose
KLKP9R	tggcagcggccgcAACATCACAGCTTCTGTTTATTAAGCA	5′ RACE, cloning
KLKP8F	tcgcagaattcATGCTGTGATTGCCATCCAGTCCCAGA	3′ RACE, cloning
KLK4r18	gatcggtcgactgACTGGCCTGGACGGTTTTCTCTAT	5′ RACE, cloning
KLKPf10	agctgaattcTAAACGGCGAGGACTGCAGCCCGCACT	cloning
KLK4Ne2f	GGCGGCACTGGTCATGGAAAACGAA	3′ RACE
KLK4Ne3f	CGGAGCATCAGCATTGCTTCGCAGT	3′ RACE
K4T2e23f	CTGCTGGCGAACGATGCTGT	qPCR
K4T2e3r	GACGTCCTTGCACTGGAAGT	qPCR
KL4e34f	CTGCTGGCGAACGGCAGAAT	qPCR
KL4e4r	GTGGTACAGCGGGTCATAGA	qPCR
KPT2e1f	ACTCCCTCTGCAGATGCTGT	qPCR
KLKPF1	CCCAACCCTGGCAGGGTTGTACCATT	DNA sequencing, cloning
KLKPR4	ACTTCGGAGAACTATGGTGCTGGCTA	DNA sequencing
KLK4Ne4r	AGCTTACTGCAGACCTCCTCAGACA	qPCR
HPRTf1	AGGGGACATAAAAGTAATTGGTGGAGAT	qPCR
HPRTr2	TGACCAAGGAAAGCAAAGTCTGCATTGT	qPCR
rpl13a-f	GTACGCTGTGAAGGCATCAA	qPCR
rpl13a-r	GTTGGTGTTCATCCGCTTG	qPCR

## Data Availability

Data are available upon request.

## Supplementary Materials

The following are available online at https://www.mdpi.com/article/10.3390/ijms222313023/s1.
