# Supplementary material for: KLK4T2 Is a Hormonally Regulated Transcript from the KLK4 Locus
Source: Int J Mol Sci. 2021 Dec 1;22(23):13023. doi: 10.3390/ijms222313023 (PMC8657796; doi:10.3390/ijms222313023)
Supplement: Supplementary file 1 [file ijms-22-13023-s001.zip › ijms-1449779-supplementary.pdf]

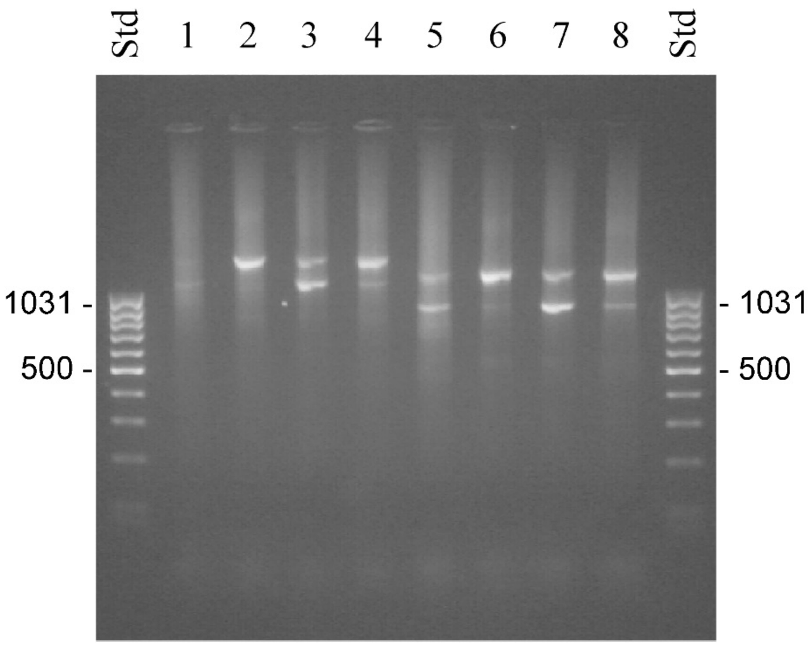

Figure S1. Transcripts detected by 3′ RACE in unfractionated BPH and compartments of LNCaP cells. RACE products were analyzed by electrophoresis in 1% agarose gels and stained by the fluorescent dye GelRed. The experiments were done with the oligonucleotides KLK4Ne2f, priming in KLK4 exon 3 (lanes 1-4) and KLK4Ne3f, priming in KLK4 exon 4 (lanes 5-8). Sources of RNA were unfractionated RNA from BPH (lanes 1 and 5) and LNCaP (lanes 2 and 6), LNCaP cytoplasm (lanes 3 and 7), and LNCaP nucleus (lane 4 and 8). MassRuler Low Range (Std) was used molecular size marker.
